# Chile: Acceptability of a Training Program for Depression Management in Primary Care

**DOI:** 10.3389/fpsyg.2016.00853

**Published:** 2016-06-06

**Authors:** Rigoberto Marín, Pablo Martínez, Juan P. Cornejo, Berta Díaz, José Peralta, Álvaro Tala, Graciela Rojas

**Affiliations:** ^1^School of Medicine, Faculty of Medicine, University of Chile, SantiagoChile; ^2^Department of Psychiatry and Mental Health, Clinical Hospital, University of Chile, SantiagoChile; ^3^School of Psychology, Faculty of Humanities, University of Santiago, Chile, SantiagoChile; ^4^Millenium Institute for Research in Depression and Personality, SantiagoChile

**Keywords:** depressive disorder, primary health care, attitude of health personnel, education, health care quality assurance

## Abstract

**Background:** In Chile, there are inconsistencies in the management of depression in primary care settings, and the National Depression Program, currently in effect, was implemented without a standardized training program. The objective of this study is to evaluate the acceptability of a training program on the management of depression for primary care health teams.

**Methods**: The study was a randomized controlled trial, and two primary centers from the Metropolitan Region of Santiago were randomly selected to carry out the intervention training program. Pre-post surveys were applied, to evaluate expectations and satisfaction with the intervention, respectively. Descriptive and content analysis was carried out.

**Result**: The sample consisted of 41 health professionals, 56.1% of who reported that their expectations for the intervention were met. All of the training activities were evaluated with scores higher than 6.4 (on a 1–7 scale). The trainers, the methodology, and the learning environment were considered strengths and facilitators of the program, while the limited duration of the training, the logistical problems faced during part of the program, and the lack of educational material were viewed as weaknesses.

**Conclusion**: The intervention was well accepted by primary health care teams. However, the clinical impact in patients still has to be evaluated.

## Introduction

In Chile, depression is one of the most common psychiatric disorders in adult population, with a six-month prevalence of 4.7% (Vicente et al., 2002) and a one-week prevalence of 5.5% (Araya et al., 2001), and it is the second leading cause of disability-adjusted life years for both sexes (Ministry of Health (Chile), 2008), making it one of the principal public health problems in the country.

The results of the 2009–2010 Chilean National Health Survey indicated that 17.2% of the general population suffered from depressive symptoms in the last year, and that 21.2% reported having been diagnosed with depression at some point in their lives (Ministry of Health (Chile), 2011). Among individuals attending primary care centers, an international study conducted by the WHO showed that the highest prevalence of depression was detected in Santiago, Chile (29.5%), while the lowest was in Nagasaki, Japan (2.6%; Goldberg and Lecrubier, 1995).

In response to the high prevalence of depression in primary care settings, international evidence has suggested the use of stepwise treatment with multiple components for management of the illness, offering different levels of complexity, and different combinations of psychosocial, pharmacological, and/or psychotherapy interventions, according to the severity of the case (Katon et al., 1996).

In line with these recommendations, a randomized controlled trial was carried out, taking into account the idiosyncrasies of the Chilean primary care system, in which professionals who are not physicians provide a large portion of the interventions in these centers. The results of this study were promising, with the active group having a recovery rate of 70% compared to a 30% rate in the control group, after 3 months; the differences between the groups were still significant at the six-month follow-up (Araya et al., 2003).

This evidence, demonstrating primary-care-based intervention to be effective, as well as cost-effective (Araya et al., 2006), was instrumental to the decision to implement a National Program for the Detection, Diagnosis, and Treatment of Depression in all primary care centers in Chile (Ministry of Health (Chile), 2000). Furthermore, due to the magnitude of this public health problem (Ministry of Health (Chile), 2008), in 2006, all individuals over the age of 15 with depression were ensured universal access to treatment, through the National Explicit Health Guarantees program (Ministry of Health (Chile), 2006).

Currently, more than 80% of depressed patients in the Chilean public health care system are treated by primary care teams (Minoletti et al., 2012). However, evaluations of the National Depression Program have identified numerous barriers impeding effective management of the condition, such as the under-estimation of depression severity, which leads to the provision of treatment that does not align with the patients’ clinical presentations, and does not corresponds to the clinical practice guidelines for depression (Alvarado et al., 2005; Alvarado and Rojas, 2011).

This situation is not limited to Chile. International evidence has also found that the management of depression is not always consistent with clinical guidelines; sub-detection of the illness, insufficient treatment, excessive use of psycho-medication, and scarce use of cognitive-behavioral interventions are fairly common occurrences (Willemse et al., 2004; Jackson et al., 2007; Lecrubier, 2007).

Because the multidisciplinary health teams in Chilean primary care settings are the gateway into the health system for a significant portion of patients, and considering that implementation of the National Depression Program does not include a training component for these health teams, the authors saw the need to train members of primary care teams to improve the detection, diagnosis, and treatment options provided to individuals with depression (Sikorski et al., 2012).

The objective of this article is to assess the acceptability of a training program in depression management for primary health care teams in Chile.

## Materials and Methods

This manuscript presents an evaluation of the acceptability of the first component of the randomized controlled trial “Comprehensive Technology-Assisted Training and Supervision Program to Enhance Depression Management in Primary Care,” corresponding to the training portion of the program. The results from the health teams of two CESFAM in the Metropolitan Region of Santiago, Chile, randomized to the study’s intervention arm, are presented.

The selection of the CESFAM for the study was carried out in two steps. First, two urban municipalities from the Metropolitan Region, with clinical campuses of the Universidad de Chile, were randomly selected. Municipalities were excluded if they had a high Human Development Index, a high percentage of immigrants, a large proportion of older adults, and/or psychiatry residents working in primary care centers. Secondly, in the two selected municipalities, two CESFAM were randomly chosen, with one assigned to the intervention arm and the other to the control arm of the study.

The training program was designed by a group of psychiatrists, psychologists, social workers, and educators from the Universidad de Chile Faculty of Medicine and Clinical Hospital.

The content of the intervention was based on recommendations from the Clinical Guidelines for Depression in Individuals older than 15 years of age (Ministry of Health (Chile), 2013) and included topics related to the detection, diagnosis, treatment, and monitoring of depression, with a multi-disciplinary perspective, considering different levels of illness severity (mild, moderate, and severe).

The training was carried out in the CCA of the Medical School of Universidad de Chile, with two, 12-h sessions held for each group. Overall, five groups were trained in April, September, and October 2014, for a total of 60 h of training in 10 sessions.

The following educational activates were included in the training: oral expositions, analysis of clinical cases, role playing to practice clinical interviewing and problem solving, and a OSCE, along with clinical simulations with standardized patients.

An open-ended survey was given to all participants before and after the training program, to explore their expectations prior to starting the program, the degree to which their expectations were met, and their perceptions of the program’s strengths and weaknesses.

In addition, immediately after the training program, a closed seven-item survey, with response options on a Likert scale (1–7, with 1 being “very bad” and 7 “very good”) was completed by the participants, to evaluate the various activities included in the program. The data from this survey were input into a 2007 Excel for Windows spreadsheet, for descriptive statistical analysis (Microsoft Corporation, 2007). The responses to the open-ended survey, on the other hand, went through a content analysis process, using Glaser and Strauss’ constant comparison method (Glaser and Strauss, 1967), to identify significant concepts, redundant portions, and differences between participating groups.

The Ethics Committee of the Faculty of Medicine, Universidad de Chile, granted approval for the study (project number 103-2012). Informed and written consents were obtained for all participants. The study was performed in accordance with the ethical standards laid down in the Declaration of Helsinki.

## Results

The sample consisted of 41 participants, who were health professionals from the selected CESFAMs (**Table 1**). All of the participants responded to the survey questions.

**Table 1:** Characteristics of participants (*n* = 41).

Characteristics		*n* (%)
Sex (female)		34 (82.9)
Profession	Physician	14 (34.1)
	Midwife	7 (17.1)
	Psychologist	8 (19.5)
	Nurse	4 (9.8)
	Social worker	6 (14.6)
	Occupational therapist	2 (4.9)

The topics, which emerged from the initial survey, suggested that the main expectations of the participants were: to improve the comprehensive management of depression (detection, diagnosis, treatment, monitoring, or referral), with a multidisciplinary approach; to learn or improve clinical interviewing skills; and to increase patients’ treatment adherence. To a lesser extent, professionals mentioned wanting to develop techniques to care for patients in crisis, and those who face multiple, contextual problems.

In terms of the degree to which these expectations were met, the post-training survey indicated that 56.1% of the professionals’ reported that their expectations were at least completely satisfied (36.6% of participants said their expectations were completely met, while 19.5% said that theirs were exceeded). On the other hand, 14.6% of the sample reported that their expectations were only partially met, 12.2% said that they did not have initial expectations, and 17.1% did not respond to that survey item.

All of the activities included in the training program were evaluated with scores higher than 6.40 (on a scale from 1 to 7). The average score was 6.63, with a standard deviation of 0.15. The professionals who most highly ranked the activities were occupational therapists, who gave an average score of 6.86, while midwives gave the lowest average score (6.43; **Table 2**).

**Table 2:** Rating of training program, by type of professional.

Professional	Average grade (standard deviation), on 1 to 7 scale
Occupational therapist	6.86 (0.23)
Physician	6.80 (0.15)
Nurse	6.57 (0.15)
Social worker	6.57 (0.15)
Psychologist	6.54 (0.11)
Midwife	6.43 (0.26)

**Table 3** shows the average evaluation scores given to each of the seven activities that formed part of the training program. The oral presentation “Components of depression treatment in primary care” was the activity most positively evaluated (6.71), and “Treatment of simulated patients” received the lowest average score (6.46).

**Table 3:** Rating of training program activities.

Activities	Average grade (standard deviation), on 1 to 7 scale
Oral presentation: components of depression treatment in primary care	6.71 (0.47)
Oral presentation: tools for the detection and diagnosis of depression in primary care	6.70 (0.50)
Analysis of clinical cases	6.66 (0.39)
Problem resolution skills	6.65 (0.44)
Role playing dynamics	6.64 (0.52)
Oral presentation: depression as a public health problem, and the National Depression Program	6.56 (0.49)
Treatment of simulated patients	6.46 (0.58)

The analysis of the health teams’ preferences reveal that the principal strengths of the program were the training staff, the methodologies used, and the educational environment, which all served to facilitate learning new skills (**Table 4**), while the short duration of the program, logistical problems with the organization of OSCE, and the lack of educational materials were seen as weaknesses (**Table 5**).

**Table 4:** Analysis of health teams’ preferences: strengths of the training program.

Aspect	Quotation (professional)
Characteristics of the training staff	*“Among the strengths, I highlight the multidisciplinary staff, who are trustworthy and open to answer all of our questions, to support our work as a team”* (Physician). *“The professors ensure the optimal development of each student”* (Midwife).
Theoretical–practical activities	*“The last evaluated activity* [treatment of simulated patients]*”* (Physician). *“The projects and practical exercises were a strength”* (Midwife). *“Strengths related to the integration of different concepts”* (Midwife).
Educational environment	*“The program was carried out in a nice, warm environment, which instilled confidence in us”* (Social worker). *“The setting of the training activities was very welcoming and encouraged interactions between members”* (Midwife).
Training program	*“An excellent space to strengthen health teams and individual professionals”* (Psychologist). *“The intense participation of health teams, to reinforce different topics that, in day-to-day practice, we don’t consider important or forget”* (Physician).
Methodology	*“The methodology”* (Psychologist). *“Participating in interactive activities, which facilitated different group interactions, was positive”* (Social assistant).

**Table 5:** Analysis of health teams’ preferences: weaknesses of the training program.

Aspect	Quotation (professional)
Duration of the program	*“I think the course was too short”* (Midwife). *“Perhaps it would be a good idea to add another session to discuss topics in separate groups, based on one’s profession”* (Physician).
Logistical problems	*“I couldn’t hear the audio well (in OSCE room)”* (Psychologist). *“Inside the consultation room, the instructions presented were for nurses… different instructions should be given based on profession of the test taker”* (Physician).
Educational material	*“The [lack of] support with educational materials could be seen as a weakness”* (Midwife).

## Discussion

This manuscript evaluates the acceptability of a novel training program in Chile on the management of depression in primary care for multidisciplinary health teams. The results of the study were promising: the training program was evaluated positively, with the participating professionals reporting a high degree of satisfaction with the quality of each component of the intervention. Furthermore, the health teams highlighted the training staff, the employed methodologies, and learning environment as particular strengths of the program that supported their skills development. Overall, the participants’ evaluation suggests that the training program was well accepted.

The content of the intervention took into account the recommendations of the Clinical Guidelines for Depression in Individuals older than 15 years of age (Ministry of Health (Chile), 2013), which was prepared by a group of experts, supported by the Ministry of Health, using the most up-to-date evidence in the field. This solid foundation for the training program should facilitate its reception and implementation in the context of health services.

It is important to consider that the human resources responsible for the management of depression in primary care services are multidisciplinary health teams, with significant operational and problem solving capacities. Thus, the training program’s use of existing human resources, and the fact that it seeks to respond to the specific educational needs of each team member, by covering concepts ranging from low-complexity interventions, such as illness detection, to those that require greater specificity and degrees of coordination, are great strengths of the intervention (Ministry of Health (Chile), 2000, 2013).

Moreover, while there are studies in the literature that incorporate patients’ or health teams’ acceptability of and/or satisfaction with depression treatments (Capoccia et al., 2004; Neumeyer-Gromen et al., 2004; Katon and Seelig, 2008; Stevenson et al., 2010; Unützer and Park, 2012), international evidence on health teams’ acceptability and/or satisfaction with the educational components of clinical trials for the management of depression is scarce, although the findings of the few existing studies are homogenous: participants evaluate training programs as acceptable, relevant, and useful for clinical practice (Stevens et al., 1997; Brown et al., 2010; Ekers et al., 2013).

In these clinical trials, though the health professionals and/or interventions vary from one study to another, the training programs are quite similar, in terms of format; the educational components are comprised of lectures and discussions to deliver theoretical knowledge, and the acquisition of clinical skills is reinforced through the use of audiovisual material and role playing (Stevens et al., 1997; Brown et al., 2010; Ekers et al., 2013).

There are, however, discrepancies observed in the results of these clinical trials. One of the studies increased the likelihood that nurses detected and correctly referred depression cases, finding significant clinical improvement (Ekers et al., 2011), and another, which tested a program with nurses who provided behavioral activation for depressed adults, achieved cost-effective results (Bruce et al., 2007). Nevertheless, an educational program based on clinical guidelines to improve illness recognition and clinical outcomes of depressed patients treated by primary care physicians failed to demonstrate these improvements (Thompson et al., 2000).

The training format used in these trials was virtually the same as the one described in the present study; none of these trials, however, included a formative OSCE as one of their educational strategies.

Other studies that did incorporate a OSCE in health team training had positive results in terms of the acquisition of clinical skills (Brown et al., 2009; Falluco et al., 2015).

In this regard, systematic reviews of clinical trials of depression management programs, which included an educational intervention, concludes that individualized training sessions—even with specific methodology—did not improve patient outcomes; in contrast, the use of complex training strategies, that consider clinical practice guidelines and feedback and actively involve health teams, achieved a moderate change in the clinical management of depression in primary care services and in patients’ symptomatology (Gilbody et al., 2003; Sikorski et al., 2012).

In light of the evidence reviewed thus far, it seems reasonable to assume that the training program described in this manuscript could result in improved depression management by participating health teams and in significant positive clinical outcomes for patients. This evaluation is pending, however, since the trial is still in the recruitment phase.

A few limitations of this study should be reviewed. First, the small sample of health professionals is a limitation, especially considering the large number of professionals and health teams working primary care centers in Chile, which are greatly heterogeneous in terms of available resources, their prioritization of resources for mental health services, and the characteristics of the populations they served, among other factors. Second, this study did not collect information on various individual or group variables with respect to the participating professionals, such as their number of years of experience working in primary care, the time they dedicate to seeing mental health patents, and their prior level of knowledge on depression, which could influence both their evaluation of the training program, and the impact of the program on patients’ clinical outcomes. Additionally, the motivation for professionals’ behavioral change was not considered for the present study, although it is a variable that has been shown to be important for the practical implementation of content covered in training programs (Shirazi et al., 2009).

The participants themselves identified two weaknesses for the training program which should be considered: (i) the short duration of the program, and (ii) the lack of educational material made available to them. In this vein, the caseload burden of primary care health teams significantly limits professionals’ time availability, open for training, though the duration of training program could be extended in the form of continuing booster sessions, to consolidate skill development, address problems in clinical practice, and cover any issues that the participants feel are pending. Regarding the second aspect, the training program should provide participants additional support, by expanding the availability and increasing the relevance of educational material.

Finally, future studies should determine not only if this depression management training program is effective in the local Chilean population, and for national public mental health policies, but also if the intervention model could be applied in primary care settings of other countries, where depression remains an unsolved public health problem.

In summary, it can be concluded that the training program was accepted by primary care health teams, and although some participants thought that certain aspects of the program could be improved, there was a high level of satisfaction with the intervention, and it coincided with the professionals’ preferences, which could indicate that its future application with other health teams could be well received. Nevertheless, investigators still have to evaluate if this training program translates into better clinical results for depressed patients, who attend Chilean primary care centers.

## Author Contributions

RM contributed to the planning of the training evaluations, the data analysis, the preparation of the manuscript and its final approval. PM collaborated with the implementation of the research project, the data analysis, the preparation of the manuscript and its final approval. JC and JP planned activities for the training program and revised the manuscript. BD participated in the execution of the training program and contributed to drafting the work. AT assisted with the manuscript, revising it critically and giving final approval for submission. GR designed and implemented the research project, carried out the training program, and reviewed critically the manuscript, giving final approval for submission.

## Conflict of Interest Statement

The authors declare that the research was conducted in the absence of any commercial or financial relationships that could be construed as a potential conflict of interest.

## Abbreviations

CCA: Center for Clinical Abilities
CESFAM: Family Health Center
OSCE: Objective Structured Clinical Examination
WHO: World Health Organization.
